# Why do I need it? I am not at risk! Public perceptions towards the pandemic (H1N1) 2009 vaccine

**DOI:** 10.1186/1471-2334-10-99

**Published:** 2010-04-19

**Authors:** Holly Seale, Anita E Heywood, Mary-Louise McLaws, Kirsten F Ward, Chris P Lowbridge, Debbie Van, C Raina MacIntyre

**Affiliations:** 1 School of Public Health and Community Medicine, Faculty of Medicine, The University of New South Wales, Sydney, Australia; 2 General Practice, NSW, Sydney, Australia; 3 Public Health Nurse, Sydney, Australia; 4 Medical Student, Faculty of Medicine, The University of New South Wales, Sydney, Australia; 5 National Centre for Immunisation Research and Surveillance of Vaccine Preventable Diseases (NCIRS), The Children's Hospital at Westmead and Discipline of Paediatrics and Child Health, University of Sydney, Sydney, Australia

## Abstract

**Background:**

On the 30th September 2009, the pandemic (H1N1) 2009 influenza vaccine was made available to adults and children aged 10 years and over, in Australia. Acceptance of a novel vaccine is influenced by perceptions of risk including risk of infection, risk of death or severe illness and risk of serious vaccine side-effects. We surveyed a sample of residents from Sydney, Australia to ascertain their risk perception, attitudes towards the pandemic and willingness to accept the pandemic (H1N1) 2009 influenza vaccine.

**Methods:**

We sampled residents using a cross-sectional intercept design during the WHO Phase 6. Members of the public were approached in shopping and pedestrian malls to undertake the survey during September and October 2009. The survey measured perceived risk, seriousness of disease, recent behavioural changes, likely acceptance of the pandemic (H1N1) 2009 vaccine and issues relating to uptake and perceived safety.

**Results:**

Of the 627 respondents, the majority felt that they had a "very low to low" (332/627, 52.9%) risk of acquiring H1N1. 24.5% (154/627) of respondents believed that the disease would "very seriously or extremely" affect their health. Nearly half (305/627, 48.6%) reported that in response to the "swine flu" outbreak they had undertaken one or more of the investigated behavioural changes. Overall, the self-reported likelihood of accepting vaccination against novel H1N1 was 54.7% (343/627).

**Conclusions:**

While, most participants did not believe they were at high risk of acquiring pandemic H1N1 2009, over half of the sample indicated that they would accept the vaccine. Participants who were vaccinated against the seasonal influenza were more likely to receive the H1N1 vaccine. Concerns about safety, the possibility of side effects and the vaccine development process need to be addressed.

## Background

In April 2009, the Mexican Secretariat of Health reported an outbreak of respiratory disease. In the affected patients, a novel swine origin influenza A (H1N1 09) virus was detected [1,2]. Evidence that this new strain could pass from human-to-human led the World Health Organization (WHO) to quickly raise its pandemic alert level to phase 5 on April 29^th^, representing "a strong signal that a pandemic is imminent and that the time to finalise the organisation, communication and implementation of the planned mitigation measures is short" [3]. After documentation of human-to-human transmission of the virus in at least three countries across two WHO regions, the WHO raised the pandemic level to 6 on June 11^th ^[4].

Australia experienced the pandemic influenza A (H1N1) 2009 outbreak at the same time that seasonal influenza circulation was expected. Victoria was the first Australian state to report a significant number of cases, followed by New South Wales [5]. The first wave lasted 18 weeks in Australia from mid-May to late September 2009. The rate of hospitalisations was 23 per 100,000 population, with indigenous Australians overrepresented [6]. The highest rate of hospitalisation occurred among children under 5 years of age [6]. As of February 19^th ^2010, there have been 37,713 confirmed cases of pandemic (H1N1) 2009 in Australia, including 191 pandemic influenza-associated deaths [7].

Since 17 June 2009, Australia's response to the pandemic has been guided by the PROTECT phase within the Australian Health Management Plan for Pandemic Influenza (AHMPPI) [8]. This newly developed phase focused on identifying and treating infection in people with moderate to severe disease and those with certain risk factors (i.e. underlying chronic diseases), controlling outbreaks in institutions and monitoring hospitalisations [9]. On September 30th 2009, the pandemic (H1N1) 2009 vaccine was made available to adults and children aged 10 years and over, in Australia. The Australian Government purchased 21 million doses of H1N1 vaccine [10]. On the basis of local safety and immunogenicity trial results the pandemic H1N1 09 influenza vaccine was registered by the Australian Therapeutic Goods Administration [11].

To assess the associations between risk perceptions of the pandemic (H1N1) 2009 influenza and intended protective behaviour changes, including willingness to be vaccinated, we carried out a community survey in Sydney, Australia. The aim of this study was to examine attitudes, concerns and behaviours around pandemic influenza (H1N1) 2009 in the general public.

## Methods

Between September 5^th ^and October 3^rd ^2009, we conducted a cross-sectional intercept survey in Sydney Australia to explore the community beliefs and risk perceptions to the influenza A (H1N1) 2009 pandemic and their attitude towards the vaccine.

### Participants and sampling

Members of the public were approached in public shopping and pedestrian malls and invited to participate in the survey. Seven geographic areas in Sydney were selected for recruitment based on socio-economically diverse populations. Four of the authors (HS/AH/KW/CL) spent two hours in each area recruiting participants at randomly chosen times of the day. During a two-hour period every fifth person was approached. The recruiter approached adults 18 years of age or older and if the recruiter was unable to determine age the participants were asked. Participants were excluded if the researcher experienced communication difficulties with them or they were not residents of Sydney. Ethics approval was obtained from the University of New South Wales, Sydney, Australia.

### Survey

Pandemic (H1N1) 2009 was referred to by its vernacular alternative "swine flu" in the survey. Four items assessed the participants perceived personal risk level and the risk level they perceived for the general community, perceived seriousness of the disease if it was contracted, and knowledge of cases of H1N1 amongst family and friends. Items measuring perception of risk and seriousness were assessed on a five point Likert-type scale. On the same scale, participants were asked to respond to the following two statements: 'In general, I think the authorities are doing a good job of dealing with the "swine flu" pandemic' and 'I do not understand what is happening with this "swine flu" pandemic'. The questions on perception of risk had been pilot tested prior to inclusion in our first community H1N1 study which was undertaken in Sydney in April 2009 [12].

Participants were asked eight items about recent influenza-related behaviours. Five items related to avoidance of places, activities or behaviours. Three items related to recommended pathogen avoidance activities; increased cleaning or disinfecting of surfaces, washing hands with soap and water more often than usual and using alcoholic hand gel more than usual. All items measured recent behaviour and were phrased "Over the past month, I have ... because of swine flu". The wording for this question was adapted with permission from a survey undertaken on influenza (H1N1) 2009 by Rubin et al [13].

Three items assessed awareness of the pandemic (H1N1) 2009 influenza vaccination, and intention for uptake while a further five items assessed the attitudes of participants towards the vaccine on a five point Likert-type scale. Sociodemographic variables included responses to gender, age, highest educational qualification, employment status, household composition, ethnicity and uptake of an annual influenza vaccine in the preceding influenza seasons (2007, 2008 and 2009). All variables used tick boxes with the exception of one open-ended question to determine reasons for acceptance or refusal of the H1N1 09 influenza vaccine

### Data analysis

The Pearson chi-square test was used to assess statistical association in univariate analyses and a p-value of < 0.05 was considered significant. Calculation of crude odds ratios (COR) and the chi-squared test were performed using EpiInfo (version 3.3.2) CDC, Atlanta, GA. During analysis, response categories were collapsed into agree, disagree or unsure. Multivariate analysis using SPSS version 17.0 (SPSS Inc. 2008) identified significant independent predictors of acceptance of a pandemic (H1N1) 2009 vaccine, calculating adjusted odds ratios (AOR) after controlling for gender, age, ethnicity, seasonal influenza vaccination in the 2008/2009 seasons, personal risk for H1N1 influenza perceived as high to very high, perceived affect on health as very to extremely affected, undertaking more than one behavioural change due to H1N1 and perception that the H1N1 situation is serious. Age, gender and educational attainment were compared with the Australian Bureau of Statistics 2006 census data for metropolitan Sydney [14] to assess representativeness to the Sydney population. Content analysis was performed on all written responses to reasons for supporting/opposing pandemic (H1N1) 2009 vaccination. Each statement was coded into a category from a list of themes developed from the data by four authors (HS, AH, KW and CL).

## Results

A total of 1458 persons were approached and inclusion criteria assessed, with 73 excluded as they were not residents of Sydney, 35 due to insufficient English proficiency and another 10 as they were <18 years of age. Of the eligible persons, 627 (47.0%) agreed to participate. Compared to Sydney residents [14], respondents were younger with 50% of survey respondents aged <35 years compared to 33% of Sydney residents and more likely to be university educated (57%) compared to 43% of Sydney residents. Demographic characteristics of the participants are listed in Table 1.

**Table 1:** Demographic characteristics of the participants

Characteristic	% (No. of participants) N = 627
**Sex**	
Male	40.7% (255)
Female	57.1% (358)
Not specified	2.2% (14)
**Age group (years)**	
18-24	23.9% (150)
25-34	26.0% (163)
35-44	16.3% (102)
45-54	13.9% (87)
55-64	12.1% (76)
≥ 65	5.3% (33)
Not specified	2.6% (16)
**Home/living arrangements**	
Live Alone	16.1% (101)
Live in shared accommodation	14.7% (92)
Live with parents	16.7% (105)
Live with partner/spouse	29.2% (183)
Live with partner/spouse and children	17.5% (110)
Other	3.3% (21)
Not specified	2.4% (15)
**Highest qualification**	
None	2.4% (15)
School certificate	5.9% (37)
High school certificate	16.4% (103)
College certificate/diploma (Tafe)	16.3% (102)
University degree/equivalent	56.9% (357)
Not specified	2.1% (13)
**Ethnic Background**	
Caucasian	67.1% (421)
Other	30.3% (190)
Not specified	2.6% (16)
**Employed**	
Working	79.3 (497)
Not working	18.2 (114)
Not specified	2.6 (16)
**Received seasonal vaccine**	
2009	28.7% (180)
2008 and 2009	21.7% (136)

Few (15.8%, 99/627) participants rated the average Sydney resident's risk of acquiring H1N1 as "very high" to "high" while the remainder rated the risk as "medium" (39.4%, 247/627) or "very low to low" (43.4%, 272/627) (Table 2). Rating of their own risk followed a similar pattern with few (17.4%, 109/627) rating it at "very high" to "high". Just under half (43.9%) of participants believed the current H1N1 situation was serious. While, just over half (52.0%, 326/627), believed they had no control over whether they got "swine flu". Most (68.7%, 431/627) had not witnessed their friends or family having "swine flu" and many also (59.8%, 375/627) believed people were still going to catch it six months time. If acquired, 61.4% of participants rated pandemic (H1N1) 2009 influenza as "somewhat affecting" their own health, while a quarter (24.5%, 154/627) thought their health would be "extremely" to "very seriously" affected. Of concern, 37.0% (232/627) of respondents did not understand what was happening with the "swine flu" pandemic.

**Table 2:** Participant risk perceptions and attitudes towards pandemic influenza (H1N1) 2009

Question	Response	(N = 627) % (n)
What level of risk do you think the average Sydney resident has of catching influenza A H1N1 or "swine flu" during this pandemic?	Very High - High	15.8% (99)
	Medium	39.4% (247)
	Very low - Low	43.4% (272)
	Unsure/Not specified	1.4% (9)
What level of risk do you think you have of catching influenza A H1N1 or "swine flu" during this pandemic?	Very High - High	17.4% (109)
	Medium	27.9% (175)
	Very low - Low	52.9% (332)
	Unsure/Not specified	1.8% (11)
If you were infected with "swine flu", how seriously do you think it would affect your health?	Not at all	7.3% (46)
	Somewhat affect	61.4% (385)
	Extremely-Very seriously affect	24.5% (154)
	Unsure/Not specified	6.7% (42)
I think the current "swine flu" situation is serious	Agree	43.9% (275)
	Disagree	37.3% (234)
	Unsure/Not specified	18.7% (117)
I do not understand what is happening with this "swine Flu" pandemic	Agree	19.9% (125)
	Disagree	63.0% (395)
	Unsure/Not specified	17.1% (107)
In general, I think the authorities are doing a good job of dealing with the "swine flu" pandemic	Agree	57.7% (361)
	Disagree	13.4% (84)
	Unsure/Not specified	29.0% (182)
I think that whether I get the "swine flu" or not is out of my control	Agree	52.0% (326)
	Disagree	33.3% (209)
	Unsure/Not specified	14.6% (92)
In my opinion, people are still going to be catching "swine flu" six months from now	Agree	59.8% (375)
	Disagree	10.4% (65)
	Unsure/Not specified	29.9% (187)
Have there been cases of "swine flu" amongst your family or friends?	Yes	27.3% (171)
	No	68.7% (431)
	Unsure/Not specified	4.0% (25)

Two or more changes to behaviour were reported in response to the swine flu situation by just under half (45.8%, 272/594) of the participants (Table 3). The most common changes included hand cleansing through increased hand washing (48.3%, 303/627) and the use of alcoholic hand gel (37.6%, 236/627). People, who reported that their risk level of acquiring "swine flu" was "high" to "very high", were 2.8 times more likely (OR 2.8, CI95 1.7-4.7, p < 0.0001) to modify their behaviours because of "swine flu".

**Table 3:** Behavioural responses to pandemic influenza (H1N1) 2009

"Over the past month, I have ... because of swine flu":	% (n) of positive responses
Washed my hands with soap and water more often than usual	48.3 (303)
	
Used alcoholic hand gel more than usual	37.6 (236)
Increased the amount I clean or disinfect things that I might touch, such as door knobs	31.1 (195)
Kept away from crowded places generally	8.6 (54)
Reduced the amount I use public transport	7.3 (46)
Deliberately cancelled or postponed a social event, such as meeting friends, eating out, or going to a sports event	6.2 (39)
Reduced the amount I go into shops	4.9 (31)
Kept one or more of my children out of school or pre-school	3.3 (21)

Over 50% (54.7%, 343/627) of the participants indicated that they intended to be vaccinated against novel H1N1 influenza. We found no association between gender or level of education and intention to receive the H1N1 vaccine. Participants from non-Caucasian ethnic groups were significantly more likely (COR 1.6, CI95 1.1-2.3, p = 0.01) to report an intention to be vaccinated with the novel H1N1 vaccine. Participants who believed they were personally at risk (rating high to very high risk of acquiring H1N1) were 1.9 times more likely (COR 1.9, CI95 1.2-3.0, p = 0.005) to intend to be vaccinated. There was no significant difference in vaccine acceptance between participants who reported cases of H1N1 amongst their friends or family members and those who did not (Table 4).

**Table 4:** Reported willingness of survey participants to accept a pandemic (H1N1) 2009 influenza vaccine

Variable	Stated acceptance of pandemic (H1N1) 2009 vaccine			
	**Yes** **(N = 343)** **% (n)**	**No/will wait N = 269** **% (n)**	**Univariate analysis** **COR (CI95) [p value]**	**Multivariate analysis** **AOR (CI95) [p value]**
Gender				
Men	40.8% (140)	39.1% (111)	1	-
Women	58.3% (200)	52.8% (150)	0.9 (0.7-1.3) [0.7]	
Not specified	0.9% (3)	2.8% (8)	-	
Age group				
18-34	53.6% (184)	43.7% (124)	1.4 (0.9-2.1) [0.2]	-
35-54	29.2% (100)	29.6% (84)	1.1 (0.7-1.7) [0.7]	
55+	16.3% (56)	18.0% (51)	1 [0.3]	
Not specified	0.9% (3)	3.5% (10)	-	
Ethnicity (Other)	35.6% (122)	23.6% (67)	1.7 (1.1-2.4) [0.01]	1.6 (1.0-2.4) [0.03] *
Received seasonal influenza vaccine in 08/09	37.6% (129)	16.9% (48)	2.7 (1.8-4.2) [<0.001]	2.7 (1.7-3.6) [<0.001]*
Personal risk†	21.3% (73)	12.0% (34)	1.9 (1.2-3.0) [0.005]	-
Undertake ≥ 1 behavioural change due to H1N1 situation	60.6% (208)	41.2% (117)	2.0 (1.4-2.8) [<0.001]	1.8 (1.2-2.5) [0.003]*
Perceives H1N1 situation as serious	55.7% (191)	27.5% (78)	3.1 (2.2-4.4) [<0.001]	2.5 (1.7-3.6) [<0.001]*

COR = Crude odds ratio; AOR = Adjusted odds ratio

†"High to very high",

* Adjusted for other variables in the model (gender, age, ethnicity, received vaccination for 2008/2009 seasons, risk for H1N1 flu perceived as high to very high, perceived affect on health as very to extremely affected,

undertaken more than one behaviour change due to H1N1 and perception that H1N1 situation is serious

Participants who received the seasonal vaccine in 2008 or 2009 were 2.7 times more likely (COR 2.7, CI95 1.8-4.0, p < 0.0001) to accept the novel H1N1 when compared with participants who had not received an annual influenza vaccine (Table 4). As expected, uptake of the 2009 seasonal influenza vaccine increased significantly with age, with vaccine uptake ranging from 20.6% (31/150) for the 18-24 age group to 60.6% (20/33) for those participants 65 years and older (p < 0.001). In comparison, participants aged 18-24 years were 1.8 times more likely (COR 1.8, CI95 1.2-2.6, p = 0.003) to indicate an intention to receive the pandemic H1N1 2009 influenza vaccine compared to older age groups. This did not remain significant on multivariate analysis (Table 4). The study participants were asked if they were concerned about vaccine safety, of which 266/627 (42%) reported that they were. A similar proportion stated that they were concerned that the vaccine had not been tested adequately (258/627, 41%). Whilst 252/627 (40%) respondents believed that the vaccine may cause people to get influenza (Table 5).

**Table 5:** Participant attitudes towards the pandemic (H1N1) 2009 influenza vaccine

Vaccination statements	Participant responses % (n)
**If a "swine flu" vaccine was made available to the general**	
**public, would you get vaccinated?**	
Yes	54.7% (343)
No	42.9% (269)
Not specified	2.3% (15)
**Only people who are have underlying medical problems or who are pregnant should be vaccinated**	
Agree	34.9% (219)
Disagree	46.3% (290)
Unsure	16.6% (104)
Not specified	2.2% (14)
**I will get the "swine flu" vaccine if the Australian government recommends it**	
Agree	52.5% (329)
Disagree	25.4% (159)
Unsure	19.8% (124)
Not specified	2.4% (15)
**I will get the "swine flu" vaccine if my doctor recommends it**	
Agree	71.5% (448)
Disagree	13.4% (84)
Unsure	12.8% (81)
Not specified	2.2% (14)
**The "swine flu" vaccine will protect me from the "swine flu"**	
Agree	44.7% (280)
Disagree	14.7% (92)
Unsure	37.8% (237)
Not specified	2.9% (18)
**I am concerned about the side effects of the "swine flu" vaccine**	
Agree	42.4% (266)
Disagree	27.6% (173)
Unsure	27.3% (171)
Not specified	2.7% (17)
**I am concerned that the vaccine has not been tested adequately**	
Agree	41.1% (258)
Disagree	24.4% (153)
Unsure	32.2% (202)
Not specified	2.2% (14)
**The "swine flu" vaccine may cause the "flu" in some people**	
Agree	40.2% (252)
Disagree	17.9% (112)
Unsure	39.6% (248)
Not specified	2.2% (15)
**The "swine flu" vaccine will stop the spread of "swine flu"**	
Agree	30.9% (194)
Disagree	24.6% (154)
Unsure	42.1% (264)
Not specified	2.4% (15)

Responses were received from 491/627 participants regarding why they would or would not accept the pandemic H1N1 2009 influenza vaccine. Close to half (49.9%, 245/491) of the responses were classified as being in support of the novel H1N1 vaccine. A common supportive reason was the belief that the vaccine would provide self protection (32.6%, 80/245), followed by the belief that the novel vaccine would provide general protection to the community and would halt the spread of the disease (31%, 76/245). Some participants classified themselves at high risk of influenza and therefore believed it was extremely important to be vaccinated, whereas others expressed an obligation to be vaccinated because of work commitments. A general belief or confidence in vaccination was commonly expressed as a reason for accepting the vaccine. Some participants believed that the novel H1N1 2009 influenza vaccine was the same as the "normal influenza vaccine" and therefore intended to be vaccinated as they would normally do against seasonal influenza.

**Figure 1:**
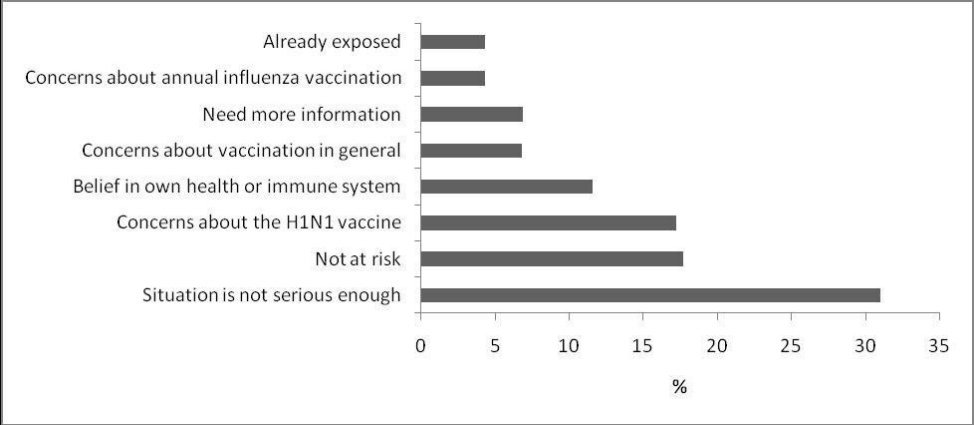
**Primary reason stated by participants for not accepting the pandemic (H1N1) 2009 influenza vaccine (N = 246)**.

The main reasons given for not accepting the new vaccine included the belief that the pandemic H1N1 2009 influenza situation was not serious enough to warrant vaccination (29.3%, 72/246,) or they did not perceive themselves to be at-risk (17.1%, 42/246) (Figure 1). Many people believed that H1N1 was just "another" influenza strain or it was just the "normal" flu. For some participants who expressed concerns, the focus related to the clinical trials conducted or the safety of the vaccine and the side-effects. Comments included a belief that vaccination for the novel H1N1 influenza strain was unnecessary because they were young and/or healthy and they believed that their immune system could deal with the virus. One male participant aged 55-64 years stated "I see no reason to treat swine flu any more seriously than flu. Maintaining a healthy lifestyle is much more important".

## Discussion

On the 18th of September 2009, Australia's independent medicines' regulator, the Therapeutic Goods Administration (TGA), announced it was to register the CSL Biotherapies pandemic (H1N1) 2009 influenza vaccine. Human adult trials had indicated that the pandemic vaccine was similar to that of the seasonal influenza vaccine with a high safety and low adverse events profile [11,15]. The registration announcement marked the commencement of the vaccination program for Australian adults. However, it also came at a time when many countries in the temperate regions of the southern hemisphere (Chile, Argentina, Australia, and New Zealand) had passed the peak of their winter influenza epidemic.

We found that less than 20% of our sample perceived that they were at significantly high risk of acquiring H1N1. This finding is comparable to the results reported in our earlier survey, which was conducted in Sydney during the WHO Pandemic Phase Five [12]. Our results are also echoed in other H1N1 studies conducted in Australia and overseas. For example, Lau et al found that only 10% of their participants (Hong Kong general public) considered themselves (10%, n = 31), their family members (10%, n = 30), or the general public (12%, n = 35) to have a high or very high chance of contracting A/H1N1 in the next year [16]. Eastwood et al, reported a slightly higher proportion (25%) of their cohort (Australian residents) perceived themselves to be at increased risk of infection [17]. It is perhaps not surprising that there is a low perception of risk amongst the community, as many of the reports on H1N1 in the newspapers and other media channels documented the virus as only causing mild influenza.

The acceptance of, and adherence to public health measures by the population depends largely on the way people perceive a threat. Pre-pandemic surveys and post-SARS studies have illustrated a dose-response relationship between the severity of pandemic and public response [18,19]. Given the reported mildness of the H1N1 2009 pandemic and overall low anxiety expressed by our surveyed participant's, low rates of behaviour change could have been expected. Surprisingly however, over half of our participants changed at least one, and commonly two, behaviours in response to the situation. This is in stark contrast with the findings of Rubin et al [13], who reported that in the early stages of the pandemic, only forty nine people (4.9%) engaged in one or more of the avoidance behaviours, and 377 (37.8%) said that they had carried out one or more of the three recommended behaviours. Whilst our participants did not believe that they were at high risk of acquiring the disease, the concerns about the consequences if caught may have been sufficient enabler to adopt relatively simple changes in behaviour.

Given that pandemic H1N1 2009 influenza was generally mild in those without risk factors, the Australian Government revised its pandemic plan to include the PROTECT phase, focusing on managing local outbreaks especially in vulnerable groups in whom disease may be serious [20]. As part of this revised plan certain measures employed at earlier stages of the national response were adjusted to ensure they supported the current situation. Although this phase continued to promote individual protection measures, such as personal hygiene, cough etiquette and voluntary isolation if symptomatic, it did not recommend the general avoidance of public places or activities. It was not surprising that few of our participants reported to keep away from public places and public transportation.

People most vulnerable to pandemic H1N1 2009 influenza infection, such as those with chronic respiratory disease, diabetes, cancer, severe obesity and conditions that suppress the immune system, as well as pregnant women and Indigenous Australians were encouraged to be the first recipients of the H1N1 vaccine. However, the vaccine was also made freely available to all Australians, through their local primary health care provider or immunisation provider. In comparison, in Australian the annual influenza vaccine is only provided free to Indigenous people aged over 50, or aged 15 to 49 who are at high risk (according to NHMRC recommendations), and all adults aged 65 years and older under the National Immunisation Program [21]. National provisional data collected in November and December 2009 by the Australian Institute of Health and Welfare showed that among the adult age groups (ages 18 to 64 years), there had only been a 14% uptake of the pandemic H1N1 2009 influenza vaccine [22]. In comparison, receipt of the vaccine was three times higher in those aged 65 years and over (42%) [22].

Given that many of our participants believed that the pandemic situation was over, it was pleasing to find that 54% of those surveyed in our study indicated a willingness to receive pandemic H1N1 2009 influenza vaccine. International studies assessing willingness to receive the pandemic H1N1 2009 influenza vaccine have reported rates that range from 36.9% (Greece [23]) to 49.6% (United States [24]). Our findings also suggest that beliefs about seasonal influenza vaccination will influence uptake of novel H1N1 vaccine. For example, we determined that (1) annual influenza recipients were significantly more likely to accept the pandemic H1N1 2009 influenza vaccine compared to their unvaccinated counterparts and (2) many participants likened the H1N1 influenza strain to being a "normal" strain of influenza, which was no more "serious" or "dangerous".

While 45% of our participants believed that the H1N1 vaccine would protect them against acquiring "swine flu", a similar proportion were concerned about the safety of the vaccine and the possibility of side effects. Common fears expressed, were that: (1) the vaccine had been "rushed through"; (2) there had been "insufficient research"; (3) the vaccine had not been "tested adequately" and (4) "long term studies" were required to ensure its "safety". This suggests that many of our participants have a lack of understanding about the process of developing seasonal influenza vaccine based on the probability of strains. While we only looked at a small subset of the population in Sydney, if these results were found to be representative, educational materials distributed about the pandemic influenza vaccine should focus on its safety record, manufacturing and the similarities between seasonal influenza vaccination and pandemic vaccine to help dispel these fears. Vaccine uptake may also be increased if General Practitioners actively promote the pandemic vaccine to their patients, given the fact that we found higher rates of compliance for physician recommended vaccination, than for government recommendation.

This study has several limitations. Firstly, we only recruited from one city of Australia. We therefore recognise the limitations of applying the results of this study to the broader Australian population. Secondly, people who could not communicate in English were excluded from the sample, which may have affected representation of ethnic minorities. Additional research is required to examine the differing reactions to the outbreak among these groups. Thirdly, as participation in our study was on a voluntary basis, this study has potential for self-selection bias by community members who are particularly concerned about pandemic influenza. Fourthly, this survey measured the samples views at a specific point in time, therefore the beliefs and attitudes reflect the information available at that time. We also did not elaborate on "requests by authorities," possibly causing participants to confuse mandatory behaviours with behaviours strongly recommended by public health authorities.

## Conclusions

While, most participants did not believe they were at high risk of acquiring pandemic H1N1 2009 influenza, over half of the sample indicated that they would accept the vaccine. Participants who were vaccinated against the seasonal influenza were more likely to intend to receive the pandemic H1N1 2009 influenza vaccine. Concerns about safety, the possibility of side effects and the vaccine development process need to be addressed.

## Competing interests

Raina MacIntyre receives funding from influenza vaccine manufacturers GSK and CSL Biotherapies for investigator-driven research. Kirsten Ward has received funding from vaccine manufacturer Wyeth to attend a conference. Neither of these payments was associated with this study. The remaining authors have no competing interests.

## Authors' contributions

HS participated in the design of the study and survey, undertook the distribution and collection, performed the analysis and drafted the manuscript. AH/MLM participated in the design of the study and survey, assisted with the analysis and reviewed the manuscript. KW/CL/DV helped perform the statistical analysis and drafted the manuscript. CRM participated in its design and coordination and helped to draft the manuscript. All authors read and approved the final manuscript.

## Pre-publication history

The pre-publication history for this paper can be accessed here:

http://www.biomedcentral.com/1471-2334/10/99/prepub
